# Catastrophic Costs among Tuberculosis-Affected Households in Egypt: Magnitude, Cost Drivers, and Coping Strategies

**DOI:** 10.3390/ijerph20032640

**Published:** 2023-02-01

**Authors:** Ramy Mohamed Ghazy, Malik Sallam, Rasha Ashmawy, Amira Mohamed Elzorkany, Omar Ahmed Reyad, Noha Alaa Hamdy, Heba Khedr, Rasha Ali Mosallam

**Affiliations:** 1 Tropical Health Department, High Institute of Public Health, Alexandria University, Alexandria 21561, Egypt; 2 Department of Pathology, Microbiology and Forensic Medicine, School of Medicine, The University of Jordan, Amman 11942, Jordan; 3 Department of Clinical Laboratories and Forensic Medicine, Jordan University Hospital, Amman 11942, Jordan; 4 Department of Clinical Research, Maamora Chest Hospital, Alexandria 21923, Egypt; 5 Egyptian Ministry of Health and Population, Alexandria 21554, Egypt; 6 Internal Medicine and Cardiology Clinical Pharmacy Department, Alexandria University Main Hospital, Alexandria 21526, Egypt; 7 Department of Clinical Pharmacy & Pharmacy Practice, Faculty of Pharmacy, Alexandria University, Alexandria 21521, Egypt; 8 MDR-TB Center, Maamora Chest Hospital, Alexandria 21912, Egypt; 9 Department of Health Administration and Behavioral Science, High Institute of Public Health, Alexandria University, Alexandria 21561, Egypt

**Keywords:** universal health coverage, patient costs, cost drivers, tuberculosis economics, health care costs, coping strategies

## Abstract

Despite national programs covering the cost of treatment for tuberculosis (TB) in many countries, TB patients still face substantial costs. The end TB strategy, set by the World Health Organization (WHO), calls for “zero” TB households to be affected by catastrophic payments by 2025. This study aimed to measure the catastrophic healthcare payments among TB patients in Egypt, to determine its cost drivers and determinants and to describe the coping strategies. The study utilized an Arabic-validated version of the TB cost tool developed by the WHO for estimating catastrophic healthcare expenditure using the cluster-based sample survey with stratification in seven administrative regions in Alexandria. TB payments were considered catastrophic if the total cost exceeded 20% of the household’s annual income. A total of 276 patients were interviewed: 76.4% were males, 50.0% were in the age group 18–35, and 8.3% had multidrug-resistant TB. Using the human capital approach, 17.0% of households encountered catastrophic costs compared to 59.1% when using the output approach. The cost calculation was carried out using the Egyptian pound converted to the United States dollars based on 2021 currency values. Total TB cost was United States dollars (USD) 280.28 ± 29.9 with a total direct cost of USD 103 ± 10.9 and a total indirect cost of USD 194.15 ± 25.5. The direct medical cost was the main cost driver in the pre-diagnosis period (USD 150.23 ± 26.89 pre diagnosis compared to USD 77.25 ± 9.91 post diagnosis, *p* = 0.013). The indirect costs (costs due to lost productivity) were the main cost driver in the post-diagnosis period (USD 4.68 ± 1.18 pre diagnosis compared to USD 192.84 ± 25.32 post diagnosis, *p* < 0.001). The households drew on multiple financial strategies to cope with TB costs where 66.7% borrowed and 25.4% sold household property. About two-thirds lost their jobs and another two-thirds lowered their food intake. Being female, delay in diagnosis and being in the intensive phase were significant predictors of catastrophic payment. Catastrophic costs were high among TB households in Alexandria and showed wide variation according to the method used for indirect cost estimation. The main cost driver before diagnosis was the direct medical costs, while it was the indirect costs, post diagnosis.

## 1. Introduction

Tuberculosis (TB) is a major public health problem with more than 90% of newly diagnosed cases and mortalities found in low-income countries [1,2,3]. TB is prevalent in low and middle-income countries because it is linked to poverty, inadequate sanitation or hygiene habits, and is easily transmitted from person to person. However, high-income nations, such as Canada, continue to report instances of tuberculosis, and it is regarded as a serious public health issue [4]. Globally, the incidence of TB reduced steeply from 184/10^5^ in 2000 to 129/10^5^ in 2020, then it showed a slight increase to reach 134/10^5^ in 2021. Similarly, the incidence of TB showed a remarkable reduction in Egypt from 26 cases per 10^5^ in 2000 to 10 per 10^5^ in 2021. This may be due to improvement in treatment success rates from 69 to 86% from 2000 to 2020 [5]. Mycobacterium tuberculosis bacilli, the causative agent of TB, have infected roughly one-third of the world’s population, putting them at a 10% lifetime chance of acquiring tuberculosis [6]. Based on clinical manifestation, tuberculosis is classified as pulmonary tuberculosis (PTB) or extrapulmonary tuberculosis (EPTB). Extrapulmonary tuberculosis (EPTB) is defined as tuberculosis affecting organs other than the lungs (e.g., pleura, lymph nodes, abdomen, genitourinary system, skin, joints and bones, or meninges) [7]. Individuals with TB are divided into two groups: those with latent TB infection (LTBI) and those with active TB illness [4]. LTBI refers to a person who has M. tuberculosis infection but the germs are not now producing active TB illness [8]. People with LTBI have no symptoms and are not infectious. Those with LTBI, on the other hand, can develop active TB illness if not treated properly. Active tuberculosis occurs when the TB bacteria grow and the individual’s immune system is impaired, resulting in infection. Depending on the individual, active tuberculosis can develop quickly or slowly after infection [4,8].

Treatment of drug-susceptible and drug-resistant M. tuberculosis strains necessitates the use of three to four antibiotics in combination, resulting in complicated drug susceptibility and resistance patterns. The M. tuberculosis strain that causes infection may be characterized as completely drug sensitive, mono-resistant, multi-drug-resistant (MDR) TB, or extended drug-resistant (XDR) TB. Individual drug susceptibility profiles influence the construction of patient-tailored treatment regimens within the latter two categories, which are subject to national norms and institutional rules [9,10]. The current drug regimen used to treat drug-susceptible TB is the result of decades of clinical trials [11]. A two-month treatment with isoniazid, rifampicin, pyrazinamide, and ethambutol, followed by four months with isoniazid and rifampicin, was initiated four decades ago and has not been modified since. In contrast, multiple drug-resistant TB clinical studies have recently concluded or are currently enrolling, creating a dynamic environment with prospects to enhance the cure rates and shorten therapy duration from 24 to 6 months [12,13]. In most countries with a high prevalence of TB, National Tuberculosis Programs (NTPs) cover the treatment cost. However, covering the cost of TB treatment has been shown to be insufficient in reducing the economic consequences of the disease, such as the non-medical costs involving transportation, accommodation, nutrition, as well as the productivity losses, which account for a substantial fraction of the economic burden for patients [14,15,16,17,18].

When out-of-pocket payment for TB exceeds a certain threshold of the total household income, negative TB outcomes follow. A study from Peru showed that out-of-pocket payments above 20% of household income were significantly associated with death, abandonment, or failure of treatment or recurrence in 2 years [19]. Another study from Indonesia found that catastrophic costs negatively affected treatment adherence at the 10 and 15% thresholds of annual household income [20]. On the other hand, financial incentives were effective both in reducing loss to follow-up and in treatment success in a study conducted in rural Nigeria [21].

The World Health Organization (WHO) tracked the economic burden for TB patients using the ‘’catastrophic cost’’ which refers to health care expenditure that exceeds a given proportion of available income [22,23]. In 2019, the WHO provisionally defined catastrophic cost as expenditure exceeding 20% of annual household income, including direct medical costs, non-medical costs, and overall indirect costs, such as lost pay and/or time away from work due to symptoms and seeking treatment [24,25].

In order to measure the percentage of direct and indirect spending of TB patients and their households, the WHO developed a tool in 2015 that measures the proportion of patients who experience catastrophic payments due to TB [26]. Countries, particularly those with a high TB burden, will be expected to conduct nationally representative TB patient cost surveys in order to establish baseline data for catastrophic costs and their main determinants, as well as to track these costs over time [26]. The findings of these surveys should also be used to help set policies for financial and social support to TB patients [25].

Following its release, several countries used the WHO tool for assessing the financial burden on TB patients. Setting a 20% of annual household income as a threshold, catastrophic costs exhibited wide variation by country where 22% of households experienced catastrophic costs in China [27], 27% in Kenya [28], 28% in South Africa [29], 32.4% in India [30], 53.1% in Uganda [31], and 60% in Myanmar [32].

In Egypt, the NTP has covered the costs of TB treatment since 2003. However, there are no social protection mechanisms that help the patient to cope with the indirect costs of treatment [33]. The present study aimed to utilize the WHO cost tool after its adaptation in an Egyptian context [34] to measure catastrophic healthcare payments among TB patients in Alexandria, Egypt. Moreover, the study looked forward to identifying the determinant of such costs. The results provide baseline data for the economic burden on TB patients in Egypt; thus, guiding the policy makers if there is a need for social protection mechanisms.

## 2. Materials and Methods

### 2.1. Sample Size and Sampling Technique

A cluster-based sample survey with stratification (number of clusters = 7) was conducted in Alexandria, Egypt in between March and June 2021. With a design effect of 1.75, an estimated catastrophic cost prevalence of 11.7% (supposing TB population size in Egypt is 8448, 600 of them are in Alexandria) [35], and a precision level of 5%, the minimal required sample size is 273.

Alexandria is divided into eight administrative regions. Each region represents a cluster. One cluster was excluded as it has no TB health facilities, thus, rendering a total of seven clusters. In each cluster, a list of diagnostic and treatment units (DTU) was obtained. Inside the health facilities, all patients who were on treatment for DS-TB or MDR-TB, and who provided consent, including children (with parental consent) were enrolled. If more than one household member was registered for treatment, costs for all the patients within a household were collected, as the impact of TB costs are analyzed at the household level. Patients were excluded if they were diagnosed with TB but did not initiate treatment. Each patient was interviewed only once and reported on expenditure retrospectively. Some patients were interviewed in the intensive treatment phase and others in the continuation treatment phase, with expenditure and time loss data collected for that phase only.

The interviewers received education on infection control and were asked to conduct the interview outdoors or in a well-ventilated separate space/room wearing an N-95 respirator mask. Patients were compensated in cash for the time and travel. The amount given was clearly stated in the information sheet (60 Egyptian pounds (EGP)).

### 2.2. WHO Survey Instrument

The WHO generic TB patient cost survey instrument gathers data from patients about their current TB treatment and costs incurred during the TB treatment phase in which they are interviewed [26].

Patients in the intensive phase of treatment were asked about pre-treatment expenses. The survey instrument gathers data on the patient’s demographic (e.g., age, gender, job status, household composition, etc.), economic status (e.g., individual and household income, household assets, household food security, etc.), direct out-of-pocket medical payments (e.g., consultation fees, laboratory tests, medication, etc.) and non-medical payments (e.g., food, accommodation, transportation), indirect costs (i.e., income lost or time lost due to TB), and caregiver costs. Patients’ estimates of nutritional supplement costs, health insurance reimbursements, social support schemes (i.e., aid and cash transfers) and TB-related societal effects were also gathered. Patients were also questioned about the financial implications of TB and how they dealt with them. This instrument was intended to be used only once per patient during their treatment [26]. Patients’ information was gathered from the TB treatment cards prior to the interview (patient name, age, sex, date of starting treatment, human immunodeficiency status (HIV)) and participants granted informed consent.

### 2.3. Calculation of Catastrophic Health Expenditure

When the human capital approach was used to calculate the indirect costs incurred by the patient, catastrophic cost was calculated as follows: total indirect cost added to total direct cost divided by the annual household income pre-TB.

When the output-related approach was used in the indirect cost calculation, the catastrophic cost was calculated as follows: (household income pre-TB–household income at survey added to total TB direct cost and divided by annual household income pre-TB). The cost was considered catastrophic if it exceeded 20% of the household income.

### 2.4. Sensitivity Analysis

The 20% threshold for catastrophic costs was varied to see how this would affect the percentage of households encountering catastrophic costs. Additional thresholds considered were 15 and 25%.

### 2.5. Operational Definitions

Direct cost calculation: direct cost refers to the out-of-pocket payment for direct healthcare costs (e.g., hospitalization, doctor office visits, medications and investigations) and direct non-health care costs (transportation, accommodation and nutritional and supplement costs) [36,37].

Indirect cost calculation: indirect cost is the cost related to the effect of illness on lost productivity. It was calculated using two approaches; the human capital, and the output approaches. In the human capital approach, an individual’s time (or loss of productive time from treatment and illness) was valued based on their estimated productive output, based on their reported income prior to being ill (by multiplying the estimated productive time lost due to treatment and illness in h by the hourly wage of the person before illness). No labor market and therefore no “market price” was considered for unemployed respondents and housewives. This approach was criticized on an equity basis as higher weights are placed on higher incomes and no weight is placed on the time of the unemployed. In the output-based calculation, loss of productivity was captured by changes in the income rather than time inputs, where change in the income during the TB episode was compared to the income prior to the TB episode. This approach accounts for the situations when days of illness or care seeking do not necessarily translate into days of lost work [36].

Annual household income pre-TB: since a considerable proportion of the Egyptian population are not part of the formal sector, the questionnaire assessed income through inquiring about household assets in addition to asking for the personal and the household income [24]. Data from the Demographic Health Survey and the Household Income and Expenditure Survey in Egypt were used to assess household assets and dwelling characteristics [28,29].

Household: is a community of individuals who share housing, finances, and at least one daily meal [38].

Diagnostic delay: is defined as a time period between a patient’s first encounter with any healthcare facility and the final diagnosis of TB; a delay is considered if this time period exceeded four weeks [26]. The time interval before diagnosis workout starts at the onset of TB symptoms till the first visit to a TB-designated facility.

The socioeconomic status (SES) of the sampled population was determined using the scale developed by Fahmy and El-Sherbini and updated by El-Gilany et al. for measurement of SES in health research in Egypt [35,39]. The scale is composed of 7 domains (education and cultural, occupation, family, family possessions, economic, home sanitation and health care) with a total score of 84, and a higher score indicating better SES. The SES is classified into very low, low, middle and high levels depending on the quartiles of the score calculated.

### 2.6. Data Analysis

The translated Arabic version was uploaded to the Open Data Kit (ODK). ODK allows the collection of data offline and uploading when online to send to the data repository. The collected data can be accessed via a computer’s web browser and utilized by the data collectors. All costs were reported in Egyptian pounds and then converted to US dollars (USD) using average currency exchange rate for the Egyptian pound during the study period (USD 1 = 15.75 Egyptian pounds). R software (V4.2, R Foundation for Statistical Computing, Vienna, Austria) was used to analyze data.

Sociodemographic and clinical predictors for encountering catastrophic cost were explored through a bivariable analysis using the odds ratio (OR). Logistic regression model was used to examine the effect of selected patient characteristics on the likelihood of encountering catastrophic cost. The dependent variable examined was catastrophic payment or not. Independent variables were age, sex, MDR, diagnostic place, TB type (PTB versus e EPTB), delay in diagnosis, treatment phase (intensive versus continuation), total TB cost, household income lost due to TB and annual household income before TB. Independent variables were all included as categorical or continuous variables in the model.

## 3. Results

About three quarters (76.4%) of the study population were males, half were in the age group of 18–35 years old, and 8.3% had MDR-TB. They were almost equally distributed between intensive and continuation phases. As for HIV status, 1.8% were HIV positive and around one fifth of the population had an unknown HIV status, and around one third encountered delay in diagnosis (29.0%). About 88.0% of patients were classified as being in a ‘’low SES’’, 18.1% were illiterate, and 17.8% were unemployed. Almost four fifths (83.7%) lived in an urban area. As for the insurance status, 80% were uninsured. The median household size was four people and the median number of rooms in the house was three (Table 1).

**Figure 1:**
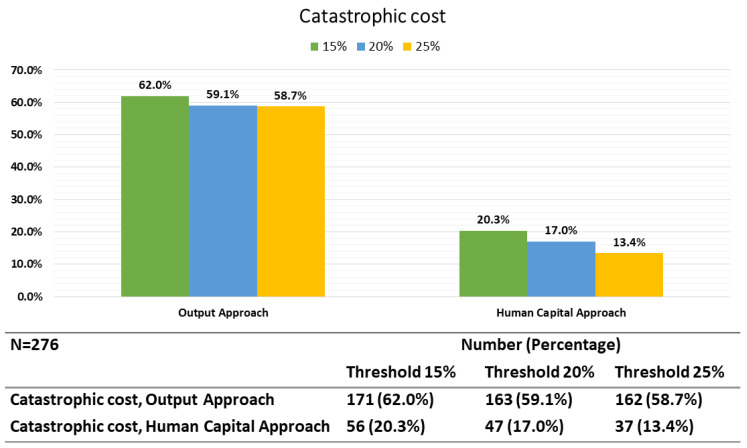
Catastrophic cost using output and human capital approaches.

Using the output approach for estimating the indirect costs, 59.1% of households encountered catastrophic costs if the total costs exceeded 20% of the household’s annual income. This percentage increased to 62.0% if a threshold of 15% (from the total household income) was used and decreased to 58.7% if a threshold of 25% was used. Using the human capital approach for indirect cost estimation, the proportion of households encountering catastrophic costs at the 20% threshold was 17.0% which increased to 20.3% using a threshold of 15% and decreased to 13.4% when a threshold of 25% was used (Figure 1).

**Figure 2:**
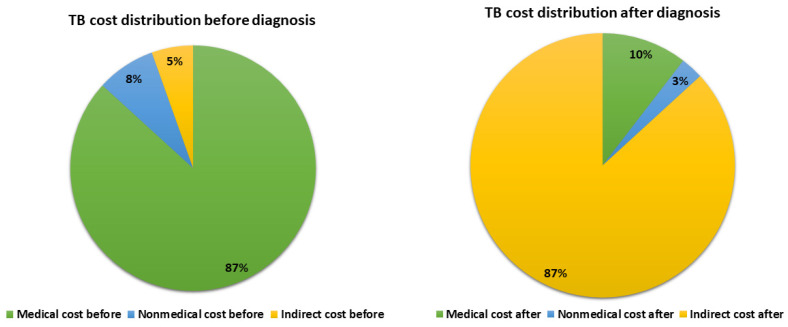
Distribution of TB costs encountered before and after diagnosis.

Total TB costs were USD 280.28 ± 29.9 with total direct costs 103 ± 10.9 and total indirect costs 194.15 ± 25.5. The direct medical costs were the main cost driver in the pre-diagnosis period (USD 150.23 ± 26.89 pre diagnosis compared to USD 77.25 ± 9.91 post diagnosis, *p* = 0.013). The indirect costs were the main cost driver in the post-diagnosis period (USD 4.68 ± 1.18 pre diagnosis compared to USD 192.84 ± 25.32 post diagnosis, *p* < 0.001). For the direct medical costs, the main cost drivers were the radiological costs and the medications with a mean of (USD 53.91 ± 13.6 and 53.76 ± 76.76, respectively). In the post-diagnosis phase, the main cost driver was the indirect cost (mean USD 194.15 ± 25.5) compared to the total direct cost (mean USD 103.0 ± 10.9). The difference between different cost drivers in the pre-diagnosis phase compared to the post-diagnosis phase was statistically significant for all items except the total direct non-medical costs. The household income was reduced from a mean of 3029.8 ± 161.39 USD pre diagnosis to a mean of 1178.0 ± 88.57 USD (Table 2, Figure 2).

Several coping strategies were used to cope with TB costs. Around one quarter of the study population used dissaving, two thirds borrowed and one third sold items (23.2, 66.7 and 34.8%, respectively). One third sold items such as household property or land (34.8%). As for the social and psychological effects, about two thirds lost their jobs and another two thirds lowered their food intake as a result of TB (66.3 and 62.0%, respectively) (Table 3).

Delay in diagnosis increased the odds of encountering a catastrophic cost. The odds of encountering catastrophic costs were 19.6 (95%CI, (6.443–59.647)) for those who had a delay of 4 or more weeks from the start of TB symptoms till diagnosis, relative to those who did not encounter such a delay. Those who were in the intensive phase had 0.108 odds (95%CI, 0.029–0.403) of encountering catastrophic costs when compared to those in the continuation phase (Table 4).

## 4. Discussion

The current study aimed to measure the catastrophic costs among TB patients in different administrative regions in the Alexandria Governorate using an adapted Arabic version of the WHO patient cost survey. Catastrophic health expenditure affected 17.0% of the study population. The main cost drivers were the direct costs in the period before diagnosis and the indirect costs during the period of treatment. Patients faced catastrophic payment by drawing on multiple coping strategies such as borrowing and selling assets. Around two thirds lost their jobs due to TB.

Catastrophic costs were much lower than those reported in multiple studies. The catastrophic costs were 33% in the Cairo governorate of Egypt [40], 32.4% in India [30], 80% in Zimbabwe [41], 53.1% in Uganda [31], and 64.1% in Ghana [42]. All studies used 20% as the threshold for calculating the catastrophic costs. However, there were differences in the methodologies for calculating the indirect costs as the numerator and the income as the denominator of the catastrophic cost. In the forementioned studies, self-reported income was used. With regard to the income, studies in Uganda and India used household expenditure for estimating the income, whereas in Zimbabwe, Egypt (Cairo), and Ghana, self-reported income prior to the TB episode was used to estimate the income. In the current study, self-reported household assets were used as the basis for calculating income [34]. Using asset linkage as the basis for estimating income is more accurate than self-reported income especially in countries with informal economies where the patient can report zero income. This was demonstrated in a South African study that used six different techniques for estimating catastrophic events. The total number of households incurring catastrophic costs ranged from 0% to 36%, depending on the estimating approach, with self-reported income much lower than the anticipated income based on asset linking.

The second source of difference in calculating the catastrophic costs in different studies is the difference in estimating the indirect costs (lost productivity). As outlined in the WHO methodology, the human capital approach and the output approach can be used in estimating the indirect cost. The first approach multiplies the time used while receiving care by the hourly wage before the TB episode, whereas the output approach values time as the difference in household income pre and post diagnosis to account for situations where illness does not translate into days of lost work. Studies using the output approach report higher percentages of catastrophic payments compared to those utilizing the human capital approach. In Uganda, Zimbabwe and Ghana, the catastrophic costs were 64.1% [31], 80.0% [41], and 53.1% [42], respectively when using the output approach compared to 33 and 32.4% in Egypt (Cairo) and India, respectively, when using the output approach and the self-reported income loss [30,40]. The difference in the catastrophic costs between both approaches is well illustrated in the present study where the human capital approach yielded 17.0% compared to 59.1% for the output approach. This outlines the importance of unifying the approach of indirect cost assessment to allow comparison between different studies and countries. The outcome based on an equity basis is usually preferred since the human capital approach places more weight on higher incomes [34].

The high percentage of catastrophic costs in the present study, whether using the human capital or outcome approaches, despite coverage of all treatment costs by the NPT in Egypt, has several interpretations. The first is that 80% of patients encountered a delay in diagnosis (> 4 weeks from the start of symptoms till being diagnosed). Additionally, patients with delayed diagnosis are 19.6 times more likely to encounter catastrophic costs compared to patients with no diagnostic delay (Table 3). Since four-fifths of the study population were uninsured, most of the care costs in the pre-diagnosis phase were paid out of pocket. This can be supported by the significantly higher direct medical costs in the pre-diagnosis phase compared to the post-diagnosis intervals (mean USD 173.8 ± 87.4 in the pre-diagnosis phase compared to USD 9.17 in the post-diagnosis phase, *p* < 0.00001). This finding agrees with that of multiple studies conducted in Egypt (Cairo), Ethiopia and India where the direct medical costs in the pre-diagnosis phase constituted a considerable proportion of the total costs [40,43,44]. This highlights the importance of governmental initiatives in the early detection and diagnosis of TB to reduce the pre-diagnosis costs. The NTP in Egypt adopted a passive case finding (PCF) with an active case finding (ACF) limited to high-risk groups as contacts of TB patients and immunosuppressed patients [45]. In a systematic review, ACF was associated with lower rates of catastrophic costs compared to PCF (12 vs. 30%, respectively) [22]. Considerations should be given to widening the criteria for ACF based on the characteristics of diagnosed patients. In addition, guidelines for how to spot the early symptoms of TB should be disseminated among the community and health professionals to allow for early detection. Further research should be conducted to explore other causes of delayed diagnosis [45].

In the current study, the indirect costs constituted 70% of the total TB costs (mean USD 194.15 from total costs of USD 280.28). These costs were mainly encountered in the post-diagnosis phase (mean USD 192.84 in the post-diagnosis phase compared to a mean of USD 4.68 in the pre-diagnosis phase) owing to lost time in treatment and hospitalization. Reduced productivity coincides with a reduction of income from a mean of USD 3029.8 before diagnosis to USD 1178.0 after diagnosis, loss of job for 66.3% of patients and using financial strategies such as selling assets and borrowing. It can also be explained by the fact that around half of the study population work in informal work with no sick leave. These results agree with those of studies in Egypt (Cairo), Ethiopia, India, Indonesia and the Philippines [40,43,44,46,47]. The high indirect economic burden which is expected to push patients into debt and poverty calls for the importance of developing TB-specific strategies such as cash transfer for households with a confirmed TB diagnosis to reimburse poor households’ costs and provide a source of income for households during TB illness, especially in families where the patient is the only breadwinner [40,48].

A considerable proportion of the study population were subject to psychological stress and some were exposed to social exclusion (22.1 and 13.8%, respectively). Studies conducted in Pakistan, India, Ethiopia, and Egypt illustrated that psychological distress in the form of depression and anxiety is common among TB patients [40,49,50,51]. Illness combined with loss of work and financial hardship are expected precursors to psychological stress. Patients exposed to psychological distress were more likely to suffer treatment failure [50,51]. In a study conducted in Ethiopia, low socioeconomic status was associated with psychological stress [51]. Since 88.0% of the study population belong to the low socioeconomic status, screening and managing this type of stress should be included in the NTP in Egypt.

At the end, ongoing efforts to diagnose cases through active, passive screening or through modern technologies [52,53] must be conducted hand in hand with catastrophic costs assessment to ensure compliance of diagnosed cases to the prescribed medications to achieve the preset goals.

### Study Limitations

This study has many strengths; first, the use of the adapted Arabic WHO cost survey questionnaire, survey design, and the use of the ODK. These helped to reduce inconsistencies in the data collected. However, it relied on patient recall, thus, it is subject to recall bias. Study participants were from the Alexandria governorate with the highest proportion living in urban areas. Thus, generalization to all Egypt, including rural residents is not possible. Another limitation is that the current study used a cross-sectional design in which the patient is interviewed only once. Costs encountered after treatment completion were not included. This is likely to underestimate the economic impact of tuberculosis. Further studies using a longitudinal design are recommended. Another point of limitation is that we did not consider the impact of coronavirus disease (COVID-19) on patient income. Patients may have been able to afford transportation to the clinic before COVID-19, but due to loss of employment of a household member, patients may not have been able to afford it after being forced to sell property to access treatment because of financial losses incurred due to COVID-19.

## 5. Conclusions

Despite free TB treatment through the NTP in Egypt, catastrophic costs are high among TB households in Alexandria with variations according to the method used for indirect cost estimation. Increased health insurance coverage and implementing more effective programs of early diagnosis and screening for TB may reduce the time till diagnosis which significantly increase the catastrophic costs incurred by patients. In addition, guidelines for how to spot the early symptoms of TB should be disseminated among the community and health professionals to allow early detection. The main cost driver before diagnosis is the direct medical costs, whereas after diagnosis it is the indirect costs. Finally, other strategies such as cash transfer for families with confirmed TB diagnosis should be considered.

## Figures and Tables

**Table 1:** Sociodemographic and clinical characteristics of the included patients.

Variable	Number (N = 276)	Percentage
**Gender (male)**	211	76.40%
**Age (years)**		
Mean (SD)	36.1 (13.5)	
<18	9	3.30%
18–35	138	50.00%
36–60	116	42.00%
>60	13	4.70%
**MDR-TB (Yes)**	23	8.30%
**TB-treatment phase**		
Continuation phase	154	55.80%
Intensive phase	122	44.20%
**HIV status**		
Negative	216	78.30%
Positive	5	1.80%
Unknown	55	19.90%
**Patient currently hospitalized (yes)**	13	4.70%
Delay in diagnosis *	80	28.99%
**Socioeconomic score**		
Median (range)	31.0 (15.0–46.0)	
**Socioeconomic status**		
Very low	28	10.10%
Low	243	88.00%
Middle	5	1.80%
High	0	0.00%
**Education level**		
Below education age	2	0.70%
Illiterate	50	18.10%
Read and write	17	6.20%
Primary	46	16.70%
Secondary	41	14.90%
Higher	120	43.50%
**Occupation**		
Unemployed/housewife	49	17.80%
Employee	95	34.40%
Hand worker	118	42.80%
Trades/business	14	5.10%
**Residence**		
Rural	33	12.00%
Slum rural	12	4.30%
Urban	231	83.70%
**Insurance status**		
No insurance	221	80.10%
Governmental insurance	34	12.30%
Private insurance	11	4.00%
Donors	10	3.60%
**Household size median, (range)**	4.0 (0–10.0) Person	
**Number of rooms median, (range)**	3.0 (1.0–8.0) Room	

* Delay in diagnosis is considered when there is more than 4 weeks from the start of symptoms till the patient is diagnosed; SD: standard deviation.

**Table 2:** Cost of illness (USD) of TB patients by cost component.

	Pre Diagnosis	Post Diagnosis	Total	*p* Value
**Household yearly income, Mean ± SD**	3029.8 ± 161.39	1178 ± 88.57	-	**<0.001**
**Total TB direct medical cost, Mean ± SD**	150.23± 26.89	77.25± 9.91	121.37 ±15.2	**0.013**
Hospitalization	6.35 ± 0.001	154.7± 59.7o	121.89 ± 47.71	<0.001
Lab tests cost	29.72 ± 7.17	12.36 ± 2.78	49.36 ± 7.62	<0.001
Radiological cost	53.91 ± 13.61	25.84 ± 4.85	76.3 ± 10.53	<0.001
Consultation	12.41 ± 1.64	4.50± 0.77	15.9 ± 1.60	<0.001
Medication	53.76 ± 12.03	9.63± 2.94	62.6 ± 10.71	<0.001
Other medical costs	6.04 ± 0.83	11.55 ± 4.26	50.4 ± 17.31	<0.001
**Total TB direct non-medical cost, Mean ± SD**	14.11 ± 2.42	19.77 ± 1.08	24.1± 1.31	**0.050**
Accommodation	3.86 ± 0.67	0.28 ± 0.14	4.7 ± 1.72	0.002
Food	16.54 ± 4.28	31.75 ± 5.92	39.49 ± 7.01	0.040
Other nonmedical	13.17 ± 2.28	4.37 ± 1.38	21.32± 3.60	0.001
**Total direct cost**	152.34 ± 59.73	48.7 ± 4.82	103 ± 10.91	**<0.001**
Total indirect cost (lost productivity) *	4.68 ± 1.18	192.84 ± 25.32	194.15 ±25.52	**<0.001**
**Total TB costs**	149.63 ± 24.6	238.04 ± 26.74	280.28 ± 29.9	**<0.001**

*p* Values calculated using paired *t*-test with statistically significant values highlighted in bold style; * Calculated using the human capital approach; SD: Standard deviation; TB: Tuberculosis.

**Table 3:** Social and psychological effects and coping strategies of study population.

Coping Strategy *	Total (N = 276)	
	N	%
**Dissaving due to TB illness**	64	23.2
**Borrowing due to TB illness**	184	66.7
**Sold any item (one or more)**	96	34.8
Sold vehicle	6	2.2
Sold household property or land	70	25.4
Sold gold	25	9.1
Use livestock	3	1.1
The social and psychological effect *		
Psychological	61	22.1
Socially excluded	38	13.8
Loss of job	183	66.3
Decreased food	171	62.0
Divorce	10	3.6

* The question allowed for multiple responses.

**Table 4:** Predictors of catastrophic payment.

Predictor	OR	95% CI	*p* Value
Intercept	14.605		0.019 *
Age	0.971	(0.939–1.004)	0.083
Sex Female^®^ Male	1 0.352	Ref (0.117–1.065)	0.064
MDR-TB No^®^ Yes	1 12.89	Ref (0.42–19.82)	0.279
Diagnosis place Chest clinic ^®^ Chest hospital Private lab Other	1 0.829 1.59 0.646	Ref (0.278–2.47) (0.11–22.96) (0.197–2.118)	0.736 0.731 0.471
TB type Extra-pulmonary TB^®^ Pulmonary TB	1 1.06	Ref (0.43–5.95)	0.481
Delay in diagnosis No ^®^ Yes	1 19.6	Ref (6.443–59.647)	<0.0001 *
Treatment phase Continuation^®^ Intensive	1 0.108	Ref (0.029–0.403)	0.001 *
Total TB cost	0.99	(0.99–1.00)	0.487
Household income lost due to TB	0.99	(0.99–1.00)	0.359
Total annual household income before TB LE	0.99	(0.99–1.00)	0.352
*p* < 0.001, R^2^ = 41.0%			

^®^ Reference category. * Statistically significant *p* values are marked with an asterisk.

## Data Availability

Dataset is available from the first author upon request.

## Abbreviations

ACF	Active case finding
PCF	Passive case finding
CI	Confidence interval
COVID-19	Coronavirus disease 2019
DTU	Diagnostic and treatment units
EPTB	Extrapulmonary tuberculosis
HIV	Human immunodeficiency virus
LTBI	Latent TB infection
MDR	Multi-drug resistance
NTP	National Tuberculosis Program
ODK	Open data kit
OR	Odds ratio
PTB	Pulmonary tuberculosis
SES	Socioeconomic status
TB	Tuberculosis
WHO	World Health Organization
XDR	Extended drug resistant
